# Contribution of the Gut Microbiota to Intestinal Fibrosis in Crohn's Disease

**DOI:** 10.3389/fmed.2022.826240

**Published:** 2022-02-07

**Authors:** Daisuke Watanabe, Nobuhiko Kamada

**Affiliations:** ^1^Division of Gastroenterology and Hepatology, Department of Internal Medicine, University of Michigan, Ann Arbor, MI, United States; ^2^WPI Immunology Frontier Research Center, Osaka University, Suita, Japan

**Keywords:** gut microbiota, Crohn's disease, animal model, adherent-invasive *Escherichia coli* (AIEC), intestinal fibrosis

## Abstract

In Crohn's disease (CD), intestinal fibrosis is a critical determinant of a patient's prognosis. Although inflammation may be a prerequisite for the initiation of intestinal fibrosis, research shows that the progression or continuation of intestinal fibrosis can occur independently of inflammation. Thus, once initiated, intestinal fibrosis may persist even if medical treatment controls inflammation. Clearly, an understanding of the pathophysiological mechanisms of intestinal fibrosis is required to diminish its occurrence. Accumulating evidence suggests that the gut microbiota contributes to the pathogenesis of intestinal fibrosis. For example, the presence of antibodies against gut microbes can predict which CD patients will have intestinal complications. In addition, microbial ligands can activate intestinal fibroblasts, thereby inducing the production of extracellular matrix. Moreover, in various animal models, bacterial infection can lead to the development of intestinal fibrosis. In this review, we summarize the current knowledge of the link between intestinal fibrosis in CD and the gut microbiota. We highlight basic science and clinical evidence that the gut microbiota can be causative for intestinal fibrosis in CD and provide valuable information about the animal models used to investigate intestinal fibrosis.

## Introduction

About a third of patients with Crohn's disease (CD) exhibit a distinct phenotype of intestinal fibrosis and stenosis over a period of 10 years (1). During the clinical course of their disease most of these patients undergo surgery or endoscopic dilation to relieve the symptoms of obstruction. Postoperative recurrence rates are high: 11–32% at 5 years, 20–44% at 10 years, and 46–55% at 20 years (2). This disease course prompted the development of several new biologics, such as vedolizumab, a monoclonal antibody against α4b7 integrin, and ustekinumab, an antibody against interleukin (IL)-12/23, in the field of CD therapy. However, mechanical treatments remain the only practical method to treat obstructive complications (3, 4). Therefore, elucidation of the cellular and molecular mechanisms of intestinal fibrosis in CD is required to improve patients' quality of life.

Why do CD patients develop intestinal fibrosis? It is a generally accepted that inflammatory bowel disease (IBD), consisting of CD and ulcerative colitis, is caused by a loss of tolerance to the gut resident bacteria, which evokes an excessive immune response in a genetically susceptible host (5). Although IBD is a multifactorial disease, human genetic studies support the association of the gut microbiota with the etiology of intestinal fibrosis. CD patients who carry mutations at the nucleotide-binding oligomerization domain 2 (NOD2) locus tend to display the fibrotic phenotype more frequently (6). The dysfunction of bacterial sensing caused by NOD2 mutations implies that intestinal fibrosis is due to the dysfunction of the recognition of the gut microbiota. In addition, several mouse models have shown that specific bacteria taxa (e.g., *Salmonella* spp.) directly induce a profibrogenic response in the gastrointestinal tract (7).

It has been shown that chronic tissue damage, impaired wound healing, and the expansion of mesenchymal cells are associated with the development of fibrosis. Multiple complex mechanisms involve several cellular components, including mesenchymal cells and immune cells. Physiologically, intestinal fibrosis is the result of an excessive accumulation of the extracellular matrix (ECM). Mesenchymal cells, such as myofibroblasts and stellate cells, serve as the main ECM producers and play a central role in the pathogenesis of fibrosis (8). Studies of cell biology have shown that microbial components affect mesenchymal cell differentiation (9), and that myofibroblasts proliferate at a faster pace in IBD patients compared to healthy individuals (10). These studies highlighted the importance of the gut microbiota in the pathophysiology of intestinal fibrosis.

On the other hand, chronic inflammation is known to be necessary for the initiation of fibrosis, based on the evidence that inflammation promotes mesenchymal differentiation, activation, and proliferation. However, an animal study using mice infected with *Salmonella enterica* serovar Typhimurium showed that eradication of the pathogen using antibiotics in the early phase of the fibrotic process did not prevent intestinal fibrosis formation (11). Despite major therapeutic advances that focus on the suppression of inflammation, the incidence rate of intestinal complications, including stricture and penetration, in CD patients has not markedly changed (12). These observations suggest that the suppression of inflammation does not simply change the clinical consequences of intestinal fibrosis in CD patients. In this context, the gut microbiota can directly activate pro-fibrotic process in myofibroblasts in addition to indirect activation of fibrotic processes through inducing inflammatory responses. However, the notion that inflammation-independent mechanisms may mediate a self-perpetuating intestinal fibrosis has not been elucidated well, leaving a knowledge gap in terms of the precise mechanisms by which the gut microbiota promotes the pathophysiology of intestinal fibrosis.

Herein, we review the insights of clinical and basic science research that link intestinal fibrosis and the gut microbiota. We highlight the cellular and molecular mechanisms by which the gut microbiota induces the formation of intestinal fibrosis.

## Clinical Evidence

### The Gut Microbiota and the Pathogenesis of Crohn's Disease

Clinically, it is known that recurrent CD can be prevented by postoperative diversion of the fecal stream (13–17). Fecal diversion surgery such as ileostomy or colostomy is indicated for patients who have advanced perianal or colorectal CD as it promotes mucosal healing and resolution of perianal disease. Most individuals who undergo fecal diversion surgery exhibit striking clinical improvement within 3–6 months, and a substantial proportion of these patients achieve remission in the long term (13–17). It has also been shown that treatment with antibiotics confers notable benefits on the clinical course of CD (18–25). These studies suggest that the eradication of certain populations of bacteria has a beneficial influence on clinical outcome for patients with CD.

Human genetic studies revealed that individuals who carry variants of the NOD2 gene are more susceptible to CD (26–28). A detailed study including disease subphenotype analysis confirmed that NOD2 has the largest effect on the development of CD and is strongly associated with ileal disease (29). Biologically, NOD2 functions as an intracellular pattern recognition receptor (PRR) for muramyl dipeptide, which is derived from peptidoglycan of both gram-positive and gram-negative bacteria (30). After intracellular stimulation by bacterial products, NOD2 activates the nuclear factor kappa B (NF-κB) pathway and provides a defensive response to protect the host from bacterial infection. A study of mice revealed that variants of the NOD2 gene directly influence intestinal inflammation and bacterial translocation. Maeda and colleagues reported that mice carrying the homozygous NOD21007fs variant have an increased activation of NF-κB after exposure to muramyl dipeptide, which increases susceptibility to bacteria-induced intestinal inflammation, thereby compromising the integrity of the intestinal barrier (31). The study of intestinal biopsies from patients with CD revealed that the presence of NOD2 variants (especially R702W and 1007fs) is associated with increased NF-κB activation and altered epithelial cell–cell contacts, leading to higher intramucosal levels of endotoxin (32). Notably, these studies indicate that a dysfunctional bacterial sensing mechanism in the host can trigger the development of CD. In addition, given that NOD2 recruits ATG16L1 to the plasma membrane, the failure to do so, as occurs in the presence of NOD2 mutants, ultimately impairs autophagosomal encapsulation of invading bacteria in dendritic cells (33, 34). In line with this function, a single nucleotide polymorphism (SNP) in ATG16L1 (re2241880, Thr300Ala) appears to be associated with an increased risk of CD (35, 36).

The introduction of culture-independent techniques to analyze 16S rRNA gene sequences facilitated a more in-depth analysis of the composition of the gut microbiota (37). It was shown that CD is associated with gut dysbiosis, a condition characterized by an imbalance between protective and harmful bacteria (38). A consistent finding of 16S rRNA gene sequencing analysis was the increase in the abundance of members of the phylum Proteobacteria (gram-negative rods, including *Escherichia* spp.) in CD patients compared with non-IBD or healthy controls, and a decrease in members of the phylum Firmicutes (gram-positive bacteria, including *Clostridium* and *Bacillus* spp.) (39). Many microbiome studies recognized the adherent–invasive strains of *Escherichia coli* (AIEC) within the *Enterobacteriaceae* family, which were often found in ileal biopsies of the patients with active CD (40, 41). Some studies suggested that the decreased abundance of the phylum Firmicutes is directly associated with the pathogenesis of CD by modulating immune functions in the intestine. Animal studies revealed that 17 strains within Clostridia clusters IV, XIVa, and XVIII can induce regulatory T cells (Tregs) in the intestine (42, 43). In addition, *Faecalibacterium prausnitzii*, which belongs to *Clostridium* cluster IV, was identified as a key player in the dysbiosis associated with ileal CD (44), and shown to produce high amounts of butyrate that has beneficial effects on IBD (45). Intriguingly, a low abundance of *F. prausnitzii* is associated with an increased risk of future flares in CD (44). Moreover, *F. prausnitzii* appears to protect the host mucosa from inflammatory injury by favoring the production of antiinflammatory cytokines, such as IL-10 (44). These data support the notion that the composition of intestinal microbiota is one of the critical factors in the pathogenesis of CD.

### The Gut Microbiota and Intestinal Fibrosis in Crohn's Disease

It has been shown that CD patients carrying a NOD2 variant, such as Arg702Trp, Gly908Arg, or the frameshift mutation Leu1007insC, are at increased risk for complications and surgery (46, 47). A metaanalysis showed that carriage of at least one NOD2 variant increased the risk of stricture in CD patients (odds ratio 1.94; 95% confidence interval 1.61–2.34) (48). Further, the presence of two NOD2 mutations predicted a 41% increase in the risk of complicated disease (i.e., the stricturing or fistulizing subtype of CD) and a 58% increase in the risk of surgery (49). These data support the idea that dysfunction of bacterial sensing by NOD2 triggers intestinal fibrosis in CD.

The increased production of microbial antibodies in serum also supports the contribution of the gut microbiota toward the pathogenesis of intestinal fibrosis in CD. The serum antibody to flagellin anti-CBir1, which reflects aberrant adaptive immunity to luminal commensal bacteria, is significantly elevated in CD patients (50). Anti-CBir1 has been shown to react with flagellins from *Clostridium* species in the gut (50). It is known that flagellins are important molecules, located on the bacterial surface and involved in both adhesion and motility (51). In addition, flagellin interacts with its toll-like receptor TLR5, leading to the activation of NF-κB and the subsequent transcriptional induction of many proinflammatory cytokines (50, 52). Dubinsky and colleagues showed that children with CD who have anti-CBir1, anti-*E. coli* outer-membrane protein C antibodies (anti-OmpC), anti-*Pseudomonas*-associated sequence I2 antibodies (anti-I2), and anti-*Saccharomyces cerevisiae* antibodies (ASCA) are at an 11-fold higher risk of developing strictures and fistulas compared to those who are seronegative (53). Also, an Irish study reported a significant association between serum anti-CBir1 positivity and a complicated disease behavior as well as ileal location (54). These results suggest that immune responses against gut microbes may contribute to the development of intestinal fibrosis.

In addition, microbiome analysis of CD patients provides information about the relationship between the gut microbiota and intestinal fibrosis. As mentioned, Sokol and colleagues reported the association of a reduced abundance of *F. prausnitzii* with an increased risk of postoperative recurrence of CD (55). Another study showed that the increased risk of CD recurrence after bowel resection was associated with enriched diversity in members of the *Enterobacteriaceae* family, and the maintenance of remission was associated with increased diversity in members of the *Lachnospiraceae* family, which reside within *Clostridium* cluster XIVa (56). Therefore, although clinical evidence of the direct influence of the gut microbiota on intestinal fibrosis has been insufficient, several studies suggest that specific microbiota contribute to the pathogenesis of intestinal fibrosis in CD.

Moreover, it is reported that intestinal myofibroblasts display different functional capacities between normal individuals and patients with IBD, particularly CD (10). Myofibroblasts isolated from CD patients proliferated faster than those derived from normal individuals and UC (10). Also, the expression patterns of TGF-β isoforms differ in CD compared to normal or UC. In CD myofibroblasts, TGF-β_3_ is significantly reduced, while TGF-β_2_ is enhanced compared to normal or UC (10). These results indicate that the differential functional capacity of myofibroblasts in CD may lead to the development of intestinal fibrosis. However, the involvement of the gut microbiota in the regulation of TGF-β isoforms remains unclear.

## Experimental Evidence that Supports the Role of Microbial Stimulation in Fibrosis

### Direct Activation of Fibroblasts Through Microbial Ligands

**Figure 1:**
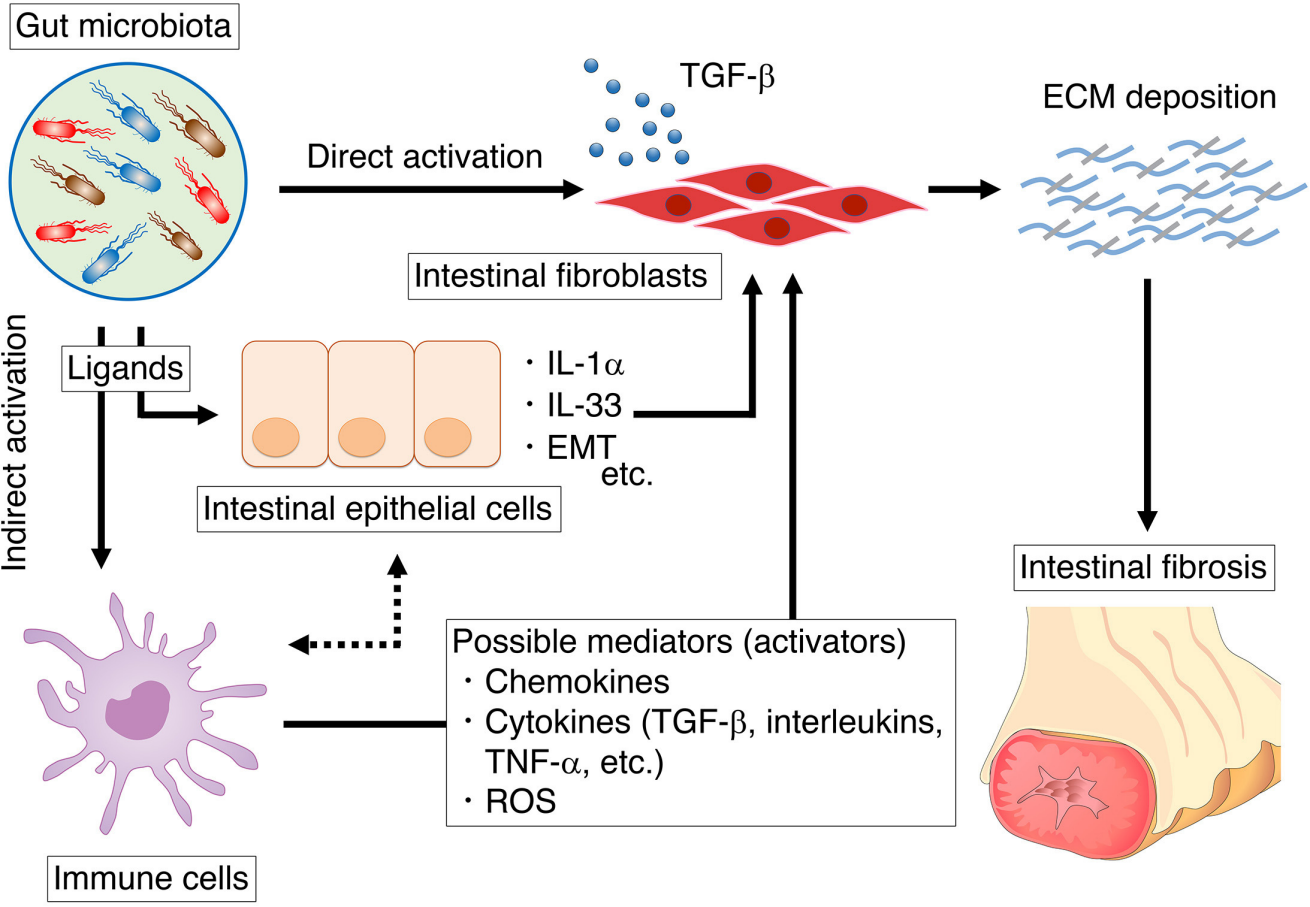
The summary of mechanisms by which the gut microbiota influences intestinal fibroblasts. We summarize the direct influence of microbiota on intestinal fibroblast (section direct activation of fibroblasts through microbial ligands). In addition, we also summarize the indirect influence of microbiota on intestinal fibroblast *via* epithelial cells or immune cells (section indirect activation of fibroblasts through the microbial ligands).

Excessive ECM synthesis is a fundamental factor in the development of fibrostenosis. In IBD, myofibroblasts originate from numerous sources, including the cells of Cajal and subepithelial myofibroblasts. It is known that bacterial components directly activate intestinal myofibroblasts (Figure 1). Activated myofibroblasts are modified fibroblasts with smooth muscle–like features and considered to be responsible for the development of intestinal fibrosis. Once activated, myofibroblasts synthesize large quantities of ECM components; primarily collagen, glycosaminoglycans, tenascin, and fibronectin (57).

It is known that microbe-derived pathogen-associated molecular patterns (PAMPs) are sensed by pattern recognition receptors (PRRs), such as toll-like receptors (TLRs) and Nod-like receptors (NLRs), expressed in intestinal immune and nonimmune cells (58). Likewise, mesenchymal cells in the intestine also express TLR1–9 and NOD1–2 (59). Among several receptors, TLR4 functions as the signaling receptor for lipopolysaccharide (LPS), the major component of the outer membrane of gram-negative bacteria (60), whereas TLR2 is activated by the cell wall components of gram-positive bacteria (61–63). Cultured intestinal myofibroblasts, once activated, secrete cytokine after TLR2 or TLR4 ligand stimulation (59). There is also evidence that intestinal fibroblasts respond to LPS by activating NF-κB signaling, which enhances collagen contraction (64).

**Figure 2:**
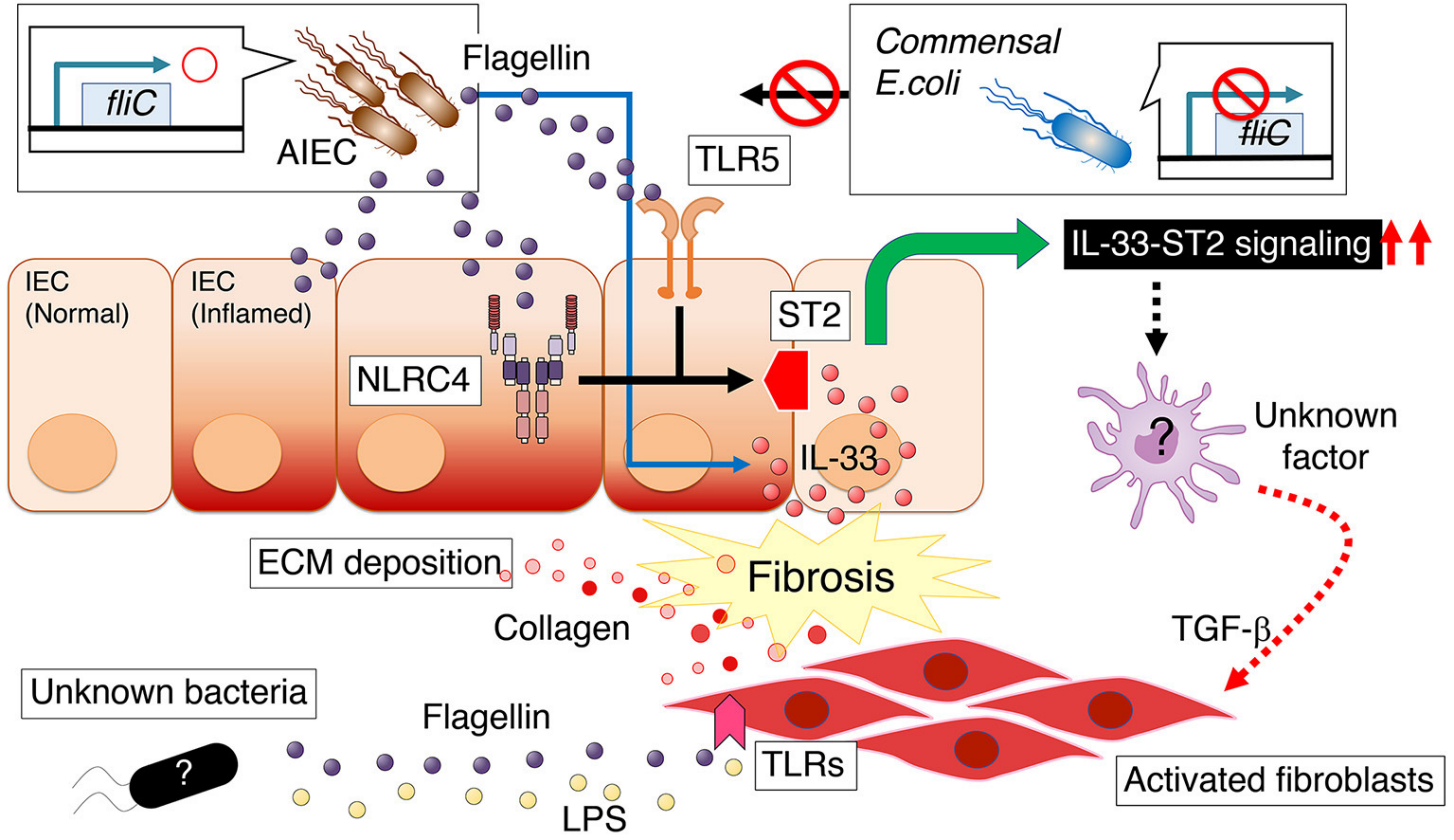
Persistent intestinal colonization of AIEC strain LF82 potentiates the development of intestinal fibrosis, a common and potentially severe complication of intestinal colitis (66). Flagellin produced by AIEC promotes the expression of interleukin 1 receptor–like 1 (IL1RL1, also known as ST2) in intestinal epithelial cells (IECs), which depends on flagellin ligands TLR5 and NLRC4 on IECs. ST2 expression augments IL-33 signaling, thereby promoting intestinal fibrosis. Conversely, there is a mechanism by which intestinal myofibroblasts directly respond to flagellin with enhanced fibronectin or collagen production in a MyD88-dependent manner (65). AIEC, adherent–invasive *Escherichia coli*; ECM, extracellular matrix; LPS, lipopolysaccharide; ST2, interleukin 1 receptor–like 1 (IL1RL1, also known as ST2).

As well as TLR2 and 4, TLR5 signaling is associated with the pathogenesis of intestinal fibrosis. Zhao et al. reported that a profibrogenic phenotype of intestinal fibroblasts is triggered exclusively by the TLR5 ligand flagellin (present in all flagellated bacteria), and this event is TGF-β1–independent and post-transcriptionally regulated (65) (Figure 2). In this study, the role of myofibroblasts to directly sense PAMPs in intestinal fibrosis was confirmed *in vivo*, as the selective deletion of MyD88 (the adaptor molecule for all TLRs except TLR3) in cells expressing α-smooth muscle actin (α-SMA) ameliorated intestinal fibrosis (65). Furthermore, the TLR5 ligand appears to promote cell cycle entry and proliferation of mesenchymal cells *in vitro* (67). Consistent with intestinal fibrosis, there is evidence that the gut microbiota promotes liver fibrosis. Elevated LPS levels have been measured in the systemic and portal circulation of patients with cirrhosis (68, 69). These studies suggest that the microbial ligand of LPS arriving from the portal vein or bacteria translocated to the liver promotes a liver fibrogenic response *via* TLR4 (68, 69). In accordance with clinical study, it has been shown that hepatic stellate cells, are activated by TLR4 ligands and mediate various fibrogenic effects (70, 71). In addition, TLR4 ligands indirectly contribute to liver fibrogenesis, rendering hepatic stellate cells more susceptible to TGF-β1 through downregulation of the TGF-β1 decoy receptor BAMBI (70). In line with the results of these studies, it has been shown that selective decontamination of the intestinal microbiota using an antibiotic agent inhibits experimental liver fibrosis with a decreased level of plasma LPS (70, 72).

### Indirect Activation of Fibroblasts Through the Microbial Ligands

In addition to intestinal fibroblasts, the gut microbiota influences immune cells and epithelial cells, which may also serve as possible cell mediators for the development of intestinal fibrosis (Figure 1). Microbial stimulation induces chemokines, cytokines, and reactive oxygen species (ROS) production by immune or epithelial cells, which in turn promote the activation of intestinal fibroblasts.

Cytokines are mediators that send a signal from a cell by binding to receptors on themselves or another cell surface. Several types of immune cells in lamina propria exert their function by producing specific cytokines, which can affect the intestinal fibroblasts. It is well known that TGF-β plays a crucial role in the machinery of intestinal mesenchymal cell activation and ECM production. The canonical TGF-β intracellular signal transduction pathway is mediated by Smad proteins as TGF-β receptor activation phosphorylates Smad2 and Smad3 and induces binding with Smad4 (73). The Smad2/3-Smad4 complex translocates into the nucleus, regulating TGF-β target genes. The Smad-dependent pro-fibrotic effects of TGF-β can result in myofibroblast activation and ECM accumulation (collagen production).

Several other cytokines are also involved in the formation of intestinal fibrosis. For instance, IL-1β can be the mediator to connect the microbiota-immune cells-intestinal fibroblasts interactions. In line with the previous studies, our laboratory showed that specific microbes accelerate IL-1β production by mononuclear cells in the lamina propria (74). It is also known that IL-1β is mainly produced by mononuclear phagocytes, acting as a pro-inflammatory effector cell of intestinal inflammation (75). In turn, IL-1β promotes the secretion of collagens I and IV, IL-8, monocyte chemoattractant protein (MCP)-1, and MMP-1 from colonic subepithelial myofibroblasts (76). These findings imply the possibility that the gut microbiota contributes to the development of intestinal fibrosis *via* induction of IL-1β from immune cells, albeit there is another contradictory report showing IL-1β inhibits collagen synthesis and induces collagenase and TIMP-1 production in intestinal smooth muscle cells (77, 78). Furthermore, TL1A, a protein encoded by TNFSF15, binds to death domain receptor 3 (DR3) and is expressed by various cell types, including immune cells. Primary intestinal myofibroblasts express DR3 and respond to TL1A, increasing collagen deposition (79). Consistently, it was reported that constitutive expression of TL1A in either lymphoid or myeloid cells leads to the acceleration of intestinal and colonic fibrosis (80, 81). Importantly, TL1A-mediated intestinal fibrosis and fibroblast activation are dependent on specific microbial populations (9). It is generally believed that Th1 cell-associated cytokines drive inflammation, whereas uncontrolled type 2 and type 17 cell responses might drive tissue fibrosis through the excessive deposition of ECM (75). IL-17 cytokines, primarily produced by Th17 cells, consists of six related proteins: IL-17A (also called IL-17), IL-17B, IL-17C, IL-17D, IL-17E (also called IL-25), and IL-17F, which signal through five receptor subunits IL-17RAIL17RE (82). It has been shown that pathogenic IL-17A-dependent immune responses are induced by microbial stimulation of DCs through NOD2, and therefore the deletion of NOD2 prevents the development of colitis (83). In addition, IL-17A enhances the production of collagen I and heat shock protein 47 (HSP47) in subepithelial myofibroblasts, which is significantly elevated in the intestinal tissues of patients with active CD (84). In line with these findings, the colonization by adherent-invasive *Escherichia coli* (AIEC) induces Th17 responses, heightens proinflammatory cytokines and fibrotic growth factors, with transmural inflammation and fibrosis (85). Previous studies have also shown that another mediator is chemokines that are leukocyte chemo-attractants that cooperate with profibrotic cytokines in fibrogenesis by recruiting myofibroblasts, macrophages, and other critical effector cells to sites of tissue injury (86). Blockade of CC- and CXC chemokine receptors decreases fibrosis in association with decreased IL-4 and IL-13 (86).

In addition to immune cells, intestinal epithelial cells also act upstream of intestinal fibroblasts, thereby contributing to intestinal fibrosis development. Epithelial cells are located at the interface of the inner lumen of the digestive tract and inside the intestinal wall. Like immune cells, epithelial cells also express various receptors for microbial ligands and produce several cytokines. For instance, IL-1α is constitutively expressed in epithelial cells, although it can be expressed by other cell types, such as macrophages, monocytes, and endothelial cells (87). Previously, it was reported that intestinal epithelial cell-derived IL-1α induces cytokine production by human intestinal fibroblasts (HIFs) (88). In addition, it has been known that IL-1α and TNF-α also increase TGF-β1 and TIMP-1 production by colonic epithelial cells (89). Also, IL-1α acts as a profibrotic cytokine in other organs, as IL-1α -deficient mice exhibit reduced collagen deposition in response to bleomycin treatment in lung fibroblasts (90).

In addition to these mechanisms, intestinal epithelial cells are involved in the pathogenesis of intestinal fibrosis *via* the machinery of epithelial-mesenchymal transition (EMT). It is well known that EMT is the primary mechanism in the development and progression of cancer and fibrosis (91). While EMT, epithelial cells change their morphology to spindle-shape, down-regulating the expression of epithelial molecules, such as E-cadherin, and gain mesenchymal characteristics, including vimentin and alpha-smooth muscle actin (α-SMA). Wang and colleagues have shown the role of the NLRP3 inflammasome in the EMT process. Interestingly, the EMT process is independent of the NLRP3 inflammasome complex formation but requires the presence of the NLRP3 protein (92).

Other mechanisms that cause intestinal fibrosis due to intestinal microbiota can be evoking oxidative stress. It has been known that polymorphonuclear leukocytes (PMNs) migrate to the site of infection or injury, engulf invading pathogens, and secrete ROS (93). ROSs are small molecules, including oxygen radicals (superoxide and hydroxyl) and non-radicals such as hypochlorous acid, singlet oxygen, and hydrogen peroxide. In the intestine, PMNs are the primary sources of ROS and reactive nitrogen species (RNS), as these cells express nicotinamide adenine dinucleotide phosphate (NADPH) oxidase enzymes (NOX/dual oxidase), the mitochondrial electron transport chain (mETC), and nitric oxide synthases (NOSs). It has also been shown that certain intestinal epithelial cells rapidly generate reactive oxygen species (ROS) in response to microbial signals (94), and subsequently, generated ROS promotes the production of several profibrogenic factors that stimulate the production or inhibit the degradation of ECM (95).

## Animal Studies

### Microbiota-Dependent Animal Models of Intestinal Fibrosis

#### Radiation-Induced Intestinal Fibrosis Mouse Model

Clinically, it is known that radiation of the small bowel and colon induces severe intestinal fibrosis. A model of radiation-induced intestinal fibrosis in rats, and to a lesser extent in mice, has been widely used to study the mechanisms of intestinal fibrosis. This model reproduces the events responsible for the intestinal fibrosis in humans observed during radiation therapy. Morphological and pathological findings in this model include radiation-induced thickening of the bowel wall, accompanied by an enlarged submucosa, increased proliferation rates of fibroblasts and smooth muscle cells, as well as enhanced accumulation of collagen and other ECM components (96). Some genetically engineered animal models of IBD do not develop intestinal fibrosis when maintained in germ-free (GF) conditions (97). Mice colonized with only *Bacteroides thetaiotaomicron* and *E. coli* have similar outcomes to the GF mice (97). Consistent with GF mice, Zhao and colleagues showed that antibiotic cocktail pretreatment before radiation effectively reduces the content of LPS and inhibits the TLR4/MyD88/NF-κB signaling pathway in the ileum (98). Antibiotic treatment also significantly improves the survival rate and attenuates intestinal injury of the mice after radiation by reducing inflammation and preventing intestinal fibrosis (98). These results indicate that the intestinal microbiota plays an important role in this model (Table 1).

**Table 1:** Summary of animal models of intestinal fibrosis associated with the gut microbiota.

**Model**	**Method**	**Site**	**Other issues**	**Ref**.
**1. Microbiota-dependent animal models of intestinal fibrosis**				
Radiation-induced intestinal fibrosis	Mice are exposed to radiation	This model depends on the site of irradiation	· Intestinal fibrosis in this model resembles the appearance in CD · Intestinal fibrosis is radioresistant to total body irradiation with 10–22 Gy · Antibiotic treatment prevents intestinal fibrosis	(97, 98)
IL-10^−/−^ mouse	Spontaneously induced	Colon, primarily Small intestine, less common	· IL-10–deficient mice housed in GF conditions fail to develop inflammation or fibrosis	(99)
SAMP1/Yit mouse	Spontaneously induced	Small bowel, primarily (terminal ileum) in early and late disease	· Intestinal histology resembles CD · SAMP1/Yit mice do not exhibit inflammation under GF conditions	(100)
TL1A overexpression-induced fibrosis	Spontaneously induced	Ileum and colon	· TL1A–Tg mice raised in GF conditions do not display an increased number or proportion of activated fibroblasts in the cecum · The major advantages of the TL1A–Tg fibrosis models are the obvious relevance to human CD	(9)
**2. Animal models of intestinal fibrosis induced by microbial components**				
PG–PS-induced intestinal fibrosis	Injection of PG–PS into the subserosa of cecal or small bowel wall	Small and large bowel	· Transmural granulomatous enterocolitis and severe transmural fibrosis in ileum and colon may be observed · Time-consuming and technically demanding technique, requiring a surgical laparotomy	(101)
Intestinal microbiota (feces)–induced intestinal fibrosis	Injection of a filtered fecal suspension into the wall of the left colon during laparotomy	Colon	· A focal and aggressive colitis with severe transmural fibrosis, elevated collagen levels, and frequent colonic strictures may be observed · Technical difficulty limits the availability of this model	(102)
**3. Animal models of intestinal fibrosis induced by bacterial infection**				
*Salmonella* spp. infection–induced intestinal fibrosis	Mice are given streptomycin orally 24 h before infection with bacteria by oral gavage	Cecum and colon	· C57BL/6 (B6) mice are extremely sensitive to wild-type *Salmonella* infection, resulting in increased mortality within the first week of infection · Use of the attenuated *S. enterica* ser. Typhimurium ΔaroA mutant causes colitis and severe fibrosis without significant mortality	(7)
AIEC (LF82) infection–induced intestinal fibrosis	Mice with DSS-injured colon (or *Salmonella* infection-injured) are orally challenged with bacteria	Colonic mucosa	· Flagellin (ligand for TLR5 or NLRC4) is necessary to exacerbate DSS-induced mouse colitis · LF82 adhesion is mediated by binding of the type 1 pili of AIEC to the host glycoprotein CEACAM6 on IECs · Flagellin produced by AIEC is a key molecule that promotes the expression of IL1RL1 in IECs, which associates with intestinal fibrosis	(66, 103–105)
AIEC (NRG857c) infection–induced intestinal fibrosis	Mice are given streptomycin orally 24 h before infection with AIEC NRG857c	Colon and cecum	· This model shows ileal and colonic inflammation that involves Th1 and Th17 immune responses · Cecal and colonic fibrosis (transmural fibrosis) in multiple mouse strains may be observed · This model shares significant similarities with CD	(85)

*AIEC, adherent-invasive Escherichia coli; CD, Crohn's disease; DSS, dextran sulfate sodium; GF, germ-free; IECs, intestinal epithelial cells; PG–PS, peptidoglycan–polysaccharide*.

#### IL-10–Deficient Intestinal Fibrosis Mouse Model

It was reported that interleukin 10 (IL-10)-deficiency aggravates intestinal (106) and renal fibrosis (107). It is well known that the interleukin 10 (IL-10)–deficient mouse shares several characteristics in common with CD (106). Nevertheless, this model has not been extensively adopted to study intestinal fibrosis. However, it has been shown that if IL-10–deficient mice undergo ileocecal resection, they develop postsurgical fibrosis in the small intestine, distant from the site of surgery, with an associated increase in procollagen-α1(I) (COL1A1) mRNA expression (99). In addition, consistent with CD patients, intestinal fibrosis develops on the proximal side of the anastomosis (108). Therefore, this model is clinically relevant and useful to study postoperative recurrence as occurs in CD. As for the machinery of intestinal fibrosis associated with IL-10 deficiency, the prohibition, which serves as a chaperone involved in stabilizing mitochondrial proteins (92), might be associated with the pathogenesis of intestinal fibrosis in IBD (109). It is well known that IL-10–deficient mice show no inflammation when housed in specific pathogen-free (SPF) conditions, but they develop intestinal inflammation on transfer to regular housing conditions (110). Interestingly, IL-10–deficient mice housed in GF conditions fail to develop inflammation or fibrosis even after ileocecal resection, suggesting that this response also depends on the presence of the gut microbiota (99) (Table 1).

#### SAMP1/Yit Intestinal Fibrosis Mouse Model

Spontaneous models of intestinal fibrosis are particularly promising because they do not depend on exogenous stimulations. The senescence accelerated mouse (SAM) P1/Yit strain was originally generated by selective breeding of the SAMP1 line (100). The SAMP1/YitFc substrain (SAMP1/Fc) was developed in Fabio Cominelli's laboratory (111) and shown to share more histomorphological features in common with human CD than the SAMP1/Yit mouse line. The SAMP1/Yit mouse develops spontaneous enteric inflammation in the ileum within 10 weeks after birth and reveals a 100% penetrance of fibrosis by 30 weeks after birth (100). In addition, a fraction of these mice spontaneously develops perianal fistulas and accumulate ECM in the small bowel and colon with thickening of the muscularis mucosa, predominantly in the terminal ileum, a feature closely resembling intestinal stricture in CD patients (111). Like the two intestinal fibrosis models just described, the SAMP1/Yit mice do not exhibit inflammation in GF conditions (100). However, the GF SAMP1/Yit mice reconstituted by transfer of the gut microbiota from SPF SAMP1/Yit mice do develop intestinal disease (100). Evidently unknown host–microbial interactions amplify the severity of intestinal disease in this model (Table 1).

#### Constitutive TL1A Expression–Induced Intestinal Fibrosis Animal Model

TL1A (a protein encoded by the TNFSF15 gene) is a member of the tumor necrosis factor (TNF) superfamily that can bind to death domain receptor 3 (DR3). A TNFSF15 haplotype appears to be associated with higher TL1A production, increased risk of CD, intestinal fibrostenosis, and greater need for surgery (112–114). In accordance with clinical data, constitutive TL1A expression in mice increases collagen deposition in the colon without detectable histologic colitis, whereas the ileum exhibits increased collagen deposition with spontaneous ileitis (80, 81, 115, 116). In addition, colitogenic conditions induced by chronic dextran sulfate sodium (DSS) treatment or adoptive T-cell transfer increase collagen deposition with fibrostenotic lesions that cause intestinal obstruction in this model (117) (Table 1).

Jacob and colleagues showed that the profibrotic and inflammatory phenotype resulting from constitutive TL1A expression is abrogated in the absence of the resident microbiota (9). Although an increased proportion of intestinal myofibroblasts can be observed in TL1A–transgenic (Tg) mice raised in conventional SPF conditions (79), TL1A–Tg mice raised in GF conditions do not display an increased number of activated fibroblasts in the cecum (9). Colonic fibroblasts isolated from the TL1A–Tg mice also displayed a significantly higher migratory capacity compared with those isolated from wild-type mice in a scratch cell migration assay; however, the enhanced rate of fibroblast gap-closure observed in TL1A–Tg mice raised in native conditions was eliminated in GF conditions (9). Furthermore, reconstitution with intestinal microbiota from SPF mice, but not human donor microbiota, resulted in increased intestinal collagen deposition and fibroblast activation in TL1A–Tg mice (9). Thus, these results indicate that TL1A-mediated intestinal fibrosis and fibroblast activation are dependent on specific microbial populations (Table 1).

#### Chemically-Induced Intestinal Fibrosis Animal Model

It has been well known that mice drinking the sugar polymer of DSS for several days develop highly reproducible colitis with bloody diarrhea, ulcerations, and weight loss (118). In the same way, chronic administration of DSS for several cycles results in intestinal fibrosis in certain strains (119). It was reported that DSS administration induces colitis in GF mice to the same extent or even more severely compared with conventionally housed mice (120), indicating that resident gut microbiota is not required for DSS-induced colitis.

However, mice develop more severe intestinal fibrosis when colonized with a pathobiont AIEC (66), suggesting that specific, most likely pathobiont-type microbiota contributes to the development of fibrosis in this model. Consistently, deletion of MyD88 results in the amelioration of intestinal fibrosis in this model (65). In contrast, a probiotic *Lactobacillus acidophilus* strain reduces the severity of DSS-induced intestinal fibrosis (121).

Likewise, the trinitrobenzene sulfonic (TNBS) acid-induced intestinal fibrosis model is one of the most commonly applied chemically-induced intestinal fibrosis models (122). Repetitive rectal TNBS application results in chronic colitis accompanied by intestinal fibrosis with luminal stenosis and bowel dilatation. TNBS administration disrupts the epithelial barrier, thereby leading to the invasion of luminal bacteria into the colonic wall in conventionally-housed animals (123). In contrast, no colitis occurs when TNBS is administered after eradication of the colonic microbiota by antibiotics (124), and some bacteria, including *Lactobacillus casei* (DN 114-001 strain), may even have protective properties in TNBS mouse model (123). Thus, the gut microbiota regulates the pathogenesis of TNBS-induced colitis and intestinal fibrosis.

### Animal Models of Intestinal Fibrosis Induced by Microbial Components

#### Animal Model of Intestinal Fibrosis Induced by Peptidoglycan–Polysaccharide

Peptidoglycan–polysaccharide (PG–PS) is a polymer composed of sugars and amino acids that is found in the bacterial cell wall. Transmural enterocolitis in rats can be observed after injection of purified sterile PG–PS derived from bacteria (e.g., *Streptococcus pyogenes*) into the subserosa of the cecal or small bowel wall during laparotomy (101). In this model, the initial insult is characterized by intense transmural inflammation and avid infiltration of acute inflammatory cells, including polymorphonuclear leukocytes. After several weeks, the acute inflammatory response becomes a patchy, chronic granulomatous inflammation, which has similarities to the chronic inflammation in CD. The affected intestinal wall becomes thickened and intraabdominal adhesions can develop (125), while areas of granulomatous inflammation express increased levels of collagen-α1 (COL1A1), TGF-β1, and IL-6 mRNA (126). Moreover, significant fibrosis and abundant mesenchymal cells surround the granulomas in this model. The mesenchymal cells have morphological and immunostaining patterns consistent with myofibroblasts, which are the key effector cells in intestinal fibrosis (126). The PG–PS model shows that the infiltration of nonviable bacterial components into the intestinal wall is sufficient to trigger inflammation and initiate intestinal fibrosis, and this infiltration can be enacted by bacterial components in the healthy intestinal lumen (Table 1).

#### Animal Model of Intestinal Fibrosis Induced by Feces and Bacterium Injection

This model shares many technical similarities to the PG–PS model. An injection of a filtered fecal suspension into the wall of the left colon of rats during laparotomy causes a focal and aggressive colitis with severe transmural fibrosis, elevated collagen levels, and frequent colonic strictures (102). A subserosal injection of a single organism suspension of intestinal anaerobes, but not aerobes, reproduces similar findings (102). The treated animals show signs of chronic inflammation and fibrosis with stricture development, significantly elevated levels of mucosal and serum TGF-β and increased collagen deposition. In this model, the increased production of TGF-β1 stimulates Smad2/3 phosphorylation and enhanced ALK5, TIMP-1, and COL1A2 gene expression (127). In addition, it has been shown that use of anti-TGF-β antibodies significantly abrogate collagen deposition in this model (102). These observations emphasize the impact of commensal intestinal bacteria on TGF-β1, collagen production, and intestinal fibrogenesis (Table 1).

#### Intestinal Fibrosis Induced by *Salmonella* spp. Infection

Nontyphoidal *Salmonella enterica* spp., such as *Salmonella enterica* serovar Typhimurium, are intestinal pathogens that can infect a wide range of animals, including humans (128, 129). It is known that certain *Salmonella* serovars are host restricted, whereas others have a broad host range. In humans, *S. enterica* ser. Typhimurium and Paratyphi can cause typhoid characterized by systemic infection, fever, and often, gastrointestinal symptoms such as diarrhea. In contrast, *S. enterica* ser. Typhimurium causes enterocolitis in humans and cattle, but systemic infection in mice (Table 1).

Serovars of *S. enterica* spp. are widely used in laboratory studies to gain an understanding of the basis of mucosal immune responses and intestinal diseases such as gastroenteritis and typhoid. It is known that oral infection with *S. enterica* ser. Typhimurium leads to spread *via* the gut-associated lymphoid tissue (GALT) to systemic sites in genetically susceptible mice. The bacteremia and lesions in the systemic organs of these mice are akin to typhoidal salmonellosis in humans; hence, this phenotype is known as mouse typhoid (130). As for *Salmonella* colonization in the mouse intestine, it is known that strain-dependent genetic susceptibility affects the host response to *Salmonella* infection. It has been shown that *S. enterica* ser. Typhimurium can cause chronic infection of systemic organs in some genetically resistant inbred mouse strains (e.g., 129SvEv, Nramp1^+/+^) (131). Consequently, these mice are useful animal models of persistent *Salmonella* systemic infection (131). On the other hand, several studies have aimed to improve *Salmonella* colonization in the mouse intestine. It is known that the intestinal tract of conventional SPF mice is poorly colonized by *S. enterica* ser. Typhimurium (~10^4^ CFU/g of contents) (132, 133). However, pretreatment of SPF mice with an antibiotic agent results in abundant colonization of *S. enterica* ser. Typhimurium in the cecum and colon, and susceptibility to colitis (132); a technique that is now widely used in this murine model of *Salmonella* spp. infection (Table 1).

In addition to studies of enterocolitis, oral administration of live *S. enterica* ser. Typhimurium has been used to study the pathogenesis of intestinal fibrosis. The severity of disease and intestinal fibrosis has been shown to depend on the genetics of the mouse strain (7). 129sv/J mice pretreated with antibiotics and chronically infected with *S. enterica* ser. Typhimurium strain SL1344 can serve as a robust model of intestinal fibrosis (7). In contrast, the C57BL/6 mouse strain is extremely sensitive to wild-type *Salmonella* infection, resulting in increased mortality within the first week of infection (7). Grassl and colleagues reported that to use C57BL/6 mice for an intestinal fibrosis model using *Salmonella* infection, the attenuated *S. enterica* ser. Typhimurium mutant strain ΔaroA (attenuated by a block in the synthesis of aromatic amino acids) can be used. These C57BL/6 mice have severe fibrosis without significant mortality (7). Thus, infection with the *S. enterica* ser. Typhimurium ΔaroA mutant allows analysis of the mechanisms that contribute to intestinal fibrosis in knockout mouse models maintained on a C57BL/6 background. So, which mouse strain and bacterial strain are the best to study intestinal fibrosis? Johnson and colleagues reported that although the severity of fibrosis in the *Salmonella* infection models varies depending on the host and bacterial strain, CBA/J mice infected with the *S. enterica* ser. Typhimurium SL1344 strain may be the optimal model for intestinal fibrosis (134) (Table 1).

This model has provided several insights that attribute the pathogenesis of intestinal fibrosis to the intestinal microbiota. First, the *Salmonella* virulence factors such as *Salmonella* pathogenicity islands (SPI)-1 and−2 are essential for the induction of intestinal fibrosis in this model (7). The resulting extensive transmural inflammation, primarily evident in the cecum but also in the colon, is accompanied by an upregulation of T helper 1 (Th1) cytokines, fibrotic growth factors, and procollagen type I. These profibrotic profiles are consistent with CD, which is associated with strong Th1 immune responses, including elevations in proinflammatory cytokine TNF-α expression. Further, early blockade of inflammation by eradicating the *S. enterica* ser. Typhimurium infection with levofloxacin ameliorates intestinal fibrosis, but does not abolish subsequent fibrosis, suggesting that once initiated, intestinal fibrosis in this model is self-propagating (11). Finally, an animal study using *Rora*^*sg*/*sg*^ BMT mice (i.e., group 2 innate lymphoid cell (ILC2)–deficient mice) showed that collagen deposition is associated with IL-17A and RORα-dependent innate lymphoid cells (ILCs) (135), affirming ILC involvement in intestinal fibrosis in this model (Table 1).

#### Intestinal Fibrosis Induced by Adherent–Invasive *E. coli*

Members of the *E. coli* family constitute a normal component of the healthy intestinal microbiota. It is known that *E. coli* strains can acquire virulence factors to adapt to harsh circumstances in the host. Many studies have reported that some *E. coli* strains isolated from the ileal lesions of CD patients exhibit adherent and invasive capabilities in gastrointestinal epithelial cells and macrophages (136–141); hence, termed adherent–invasive *Escherichia coli* (AIEC) (136). Cell biological studies showed that AIEC phagocytosed by macrophages are more resistant to xenophagy and capable of inducing a persistent inflammatory response by releasing large amounts of TNF-α (141, 142). Interestingly, monocytes from CD patients who carry homozygous or heterozygous NOD2 polymorphisms display reduced secretion of IL-1β, IL-6, and IL-10 after AIEC infection *in vitro* compared with monocytes from CD patients without NOD2 polymorphisms (143). In addition, clinical studies showed that AIEC strains are preferentially observed in ileal CD (40, 137, 144).

To date, there are two well-characterized prototypic AIEC strains: LF82 and NRG857c (85, 136, 145). The prototype AIEC strain LF82 colonizes the intestinal mucosa and induces proinflammatory cytokines during acute DSS-induced colitis (103–105). In this model, flagellin (ligand for TLR5 and NLRC4) is necessary for the AIEC strain LF82 to exacerbate DSS-induced mouse colitis, while the nonflagellated LF82 mutant strain behaves like the nonpathogenic *E. coli* strain K12 (103). Mechanistically, it has been shown that its adhesion is mediated by binding of the type 1 pili of AIEC to the host glycoprotein carcinoembryonic antigen–related cell adhesion molecule (CEACAM) 6 on the intestinal epithelial cells (IECs) (104, 146). Barnich and colleagues reported that CD patients with ileal disease have an abnormal ileal expression of CEACAM5 and 6, and that only CEACAM6 acts as a receptor for AIEC (146). Intriguingly, *in vitro* studies demonstrated that CEACAM6 expression is increased in cultured IECs after infection with AIEC, indicating that AIEC promotes its own colonization through induction of CEACAM6 expression in the host (146). In addition to these mechanisms, it has been shown that bacterial adhesion to IECs is mediated *via* chitin-binding domains in bacteria, encoded by bacterial chitinase ChiA, that interact with human chitinase CHI3L1 expressed on IECs in inflammatory conditions (105) (Table 1).

As well as LF82, a human CD isolate of AIEC strain NRG857c was used to develop a chronic AIEC infection mouse model to study intestinal inflammation and fibrosis (85). Like the *S. enterica* ser. Typhimurium infection model, mice were pretreated with oral streptomycin prior to infection with NRG857c (85). After NRG857c infection, this model showed that ileal and colonic inflammation involves Th1 and Th17 immune responses (85). The resulting inflammation leads to cecal and colonic fibrosis in multiple mouse strains, in varying degrees, and progresses to transmural fibrosis (85). This model shares significant similarities with CD (Table 1).

Our laboratory has shown that persistent intestinal colonization of AIEC strain LF82 potentiates the development of intestinal fibrosis in conditions of *Salmonella*-induced or DSS-induced colitis (66) (Figure 2). In this model, flagellin produced by AIEC, a principal component of bacterial flagella, is a key molecule that promotes the expression of interleukin 1 receptor–like 1 (IL1RL1, also known as ST2) in intestinal epithelial cells (IECs), which depends on flagellin ligands TLR5 and NLRC4 on IECs (66). Further, it has been shown that ST2 expression in IECs augments IL-33 signaling, thereby promoting intestinal fibrosis, as the blockade of IL-33–ST2 signaling by anti-ST2 antibody significantly ameliorates intestinal fibrosis (66). Therefore, therapeutic approaches that target AIEC or its downstream IL-33–ST2 signaling pathway would benefit CD patients with intestinal fibrosis (Table 1).

## Microbiota-Targeted Therapy for Intestinal Fibrosis

Accumulating evidence suggests that interventions against the gut microbiota may regulate the prognosis of intestinal fibrosis in CD. However, we have not had any therapeutical options using microbiota-targeted interventions that specifically treat intestinal fibrosis (147). Although we have various strategies that modulate the gut microbiota (e.g., antibiotics, probiotics, fecal microbiota transplantation [FMT]), it is challenging to evaluate the effects of interventions on intestinal fibrosis. This is due to the unavailability of quantification methods for intestinal fibrosis. Albeit several diagnostic tools, such as ultrasound, computer tomography, magnetic resonance, and gastrointestinal endoscopy, are available to estimate the developmental status of intestinal fibrosis in CD, there are no modalities to quantify the degree of intestinal fibrosis without conducting the surgical resection of the affected intestine (147). In addition, because intestinal fibrosis gradually progresses in CD in several decades, a much longer time should be required to certificate its effectiveness of action. Another possible reason is that it is also challenging to target pathobionts selectively without affecting other bacteria in the healthy intestine. Although antibiotics have provided significant advances in therapies for infectious diseases, several bacterial species susceptible to the agents will be affected by the treatment.

On the other, there are some success stories of “microbiota-targeted therapy” in fields other than intestinal fibrosis. For instance, several researchers reported that FMT could be a promising treatment to induce remission in UC with active disease (148, 149). In addition, probiotics may be the candidate for the treatment because it has suppressed UC relapse (150). These data might indicate that microbiota-targeted therapy is promising, and therefore we hope that practical application of microbiota-targeted therapies for intestinal fibrosis come on the stage in the near future.

## Conclusion

The cellular and molecular mechanisms of intestinal fibrosis are the focus of intense investigation. Clearly, patients who suffer from intestinal fibrosis need novel and more effective treatments that target this common, often severe, complication of CD. Compared to the enormous advances in the development of new therapies to control intestinal inflammation, such as anti-TNFs, anti-integrins, and kinase inhibitors, progress to develop therapeutic modalities that may prevent or reverse intestinal fibrosis in CD is limited. As reviewed, the gut microbiota may have a considerable impact on the pathophysiology of intestinal fibrosis in CD. Of note, several animal models enable the investigation of the precise role of the gut microbiota in the development of intestinal fibrosis. Also, technical advances provide access to the global data associated with the alterations of gene expression and gut microbial composition during the process of intestinal fibrosis. These research tools may identify specific microbes or microbial components and virulence factors that affect intestinal fibrosis development. A rational identification of microbes and microbial factors could lead to effective therapies for preventing and attenuating intestinal fibrosis.

## Author Contributions

DW and NK wrote the manuscript and approved it for publication.

## Funding

This work was supported an Overseas Postdoctoral Fellowship from the Uehara Memorial Foundation and a Postdoctoral Fellowship for Research Abroad from the Japan Society for the Promotion of Science (to DW) and National Institutes of Health grants DK108901, DK119219, AI142047, and DK125087 (to NK).

## Conflict of Interest

The authors declare that the research was conducted in the absence of any commercial or financial relationships that could be construed as a potential conflict of interest.

## Publisher's Note

All claims expressed in this article are solely those of the authors and do not necessarily represent those of their affiliated organizations, or those of the publisher, the editors and the reviewers. Any product that may be evaluated in this article, or claim that may be made by its manufacturer, is not guaranteed or endorsed by the publisher.
